# Editorial on the Special Issue “Advanced Hydrogels for the Repair of Cartilage Defects”

**DOI:** 10.3390/gels9010006

**Published:** 2022-12-22

**Authors:** Wei Wei

**Affiliations:** 1 Department of Orthopaedic Surgery, The Fourth Affiliated Hospital, International Institutes of Medicine, Zhejiang University School of Medicine, Yiwu 322000, China; zjewwei@zju.edu.cn; 2 Key Laboratory of Tissue Engineering and Regenerative Medicine of Zhejiang Province, Zhejiang University School of Medicine, Hangzhou 310000, China

This Special Issue focuses on the recent advances in orthopedic hydrogels and regenerative medicine for the repair of cartilage defects. Hydrogels are a class of water-containing biomaterial consisting of three-dimensional polymer networks. Research on biomedical hydrogels has grown fast over the years. Not only have scientists in materials science contributed to innovations but chemists, engineers, and clinical doctors have also collaborated. A variety of sources have been used to design advanced hydrogels for tissue engineering and regeneration medicine. Advanced hydrogels have biomimetic components, tunable mechanical properties, and various forms to enhance cell proliferation and deliver cells to the defect sites for tissue regeneration. This Special Issue provides high-quality research articles and reviews in advanced hydrogels for orthopedic tissue engineering and regenerative medicine.

PVA-based hydrogels hold satisfactory biocompatibility and chemical stability for cartilage replacement. However, the lack of therapeutic effects limits its practical applications. Branco et al. developed a new PVA-based hydrogel loading with diclofenac for cartilage repair [1]. The obtained hydrogels with PEG immersion showed improved tribomechanical properties. The annealing treatment endowed the hydrogel with a low friction coefficient and ensured the controlled release of diclofenac. For the drug delivery, injectable hydrogel could be a more attractive candidate due to its non-traumatic feature. García-Couce et al. designed a thermosensitive hydrogel based on chitosan and Pluronic F127 for the delivery of dexamethasone [2]. Tripolyphosphate was used as a crosslinker and was found to be able to modify the morphology of the hydrogel. The hydrogel showed a superior sustained release profile compared to that of pure Pluronic F127 hydrogel and was demonstrated to prolong the retention time of molecules in vivo. Intra-articular administration of anti-inflammatory drugs could be a way to treat osteoarthritis.

Cartilage tissue engineering using stem cells or chondrocytes is a promising strategy for the treatment of cartilage defects in osteoarthritis. Stem cells possess both multipotency for tissue regeneration and immunomodulatory effects to alleviate inflammation. However, the application of stem cells is limited by the large-scale production and viability maintenance in the target site. Hydrogels could be proper carriers for stem cells to solve the abovementioned problems. Liu et al. summarized recent approaches of hydrogel-enhanced stem-cell therapy for cartilage repair in osteoarthritis [3]. This review provides a perspective for developing advanced hydrogels to address the challenges in stem-cell-based therapy. Microgels and dynamic hydrogels were particularly discussed. Gene delivery and genome editing assisted by hydrogels for cartilage repair in osteoarthritis are highlighted. In a specific study, Teng et al. used a microfluidic system to produce hydrogel microspheres for the proliferation and delivery of stem cells [4]. The hydrogel microspheres consisting of gelatin support the adhesion of stem cells in a three-dimensional condition. Stem cells proliferated on the hydrogel microspheres and were delivered to the defect site for tissue regeneration. In another study, Wang et al. loaded chondrocyte spheroids in a gelatin methacrylate/hyaluronic acid methacrylate hybrid hydrogel to enhance the proliferation and phenotype maintenance [5]. They found that chondro-spheroid-laden hydrogel had more cartilage-related gene expression and matrix secretion both in vitro and in vivo. This approach provided a new insight into cartilage tissue engineering.

Taken together, this Special Issue on Advanced Hydrogels for the Repair of Cartilage Defects provides a glimpse into recent innovations in orthopedic hydrogel materials. Nowadays, the treatment of cartilage defects in osteoarthritis is still a clinical challenge. Advanced materials need to be developed for the replacement or regeneration of articular cartilage. The interactions between hydrogels and stem cells/chondrocytes should be taken into consideration and investigated in depth. Highly biocompatible hydrogels are expected to be developed for better translation in the clinic to help patients earlier. We hope this Special Issue paves the way for more original ideas for both advanced hydrogels and cartilage tissue engineering.
